# Diet patterns and risk of sepsis in community-dwelling adults: a cohort study

**DOI:** 10.1186/s12879-015-0981-1

**Published:** 2015-06-14

**Authors:** Orlando M. Gutiérrez, Suzanne E. Judd, Jenifer H. Voeks, April P. Carson, Monika M. Safford, James M. Shikany, Henry E. Wang

**Affiliations:** Departments of Medicine, University of Alabama at Birmingham, ZRB 614, 1720 2nd AVE S, Birmingham, AL 35294-0006 USA; Departments of Epidemiology, University of Alabama at Birmingham, Birmingham, AL USA; Departments of Biostatistics, University of Alabama at Birmingham, Birmingham, AL USA; Departments of Emergency Medicine, University of Alabama at Birmingham, Birmingham, AL USA; Department of Neurosciences, Medical University of South Carolina, Charleston, SC USA

**Keywords:** Diet patterns, Sepsis, Epidemiology

## Abstract

**Background:**

Sepsis is the syndrome of body-wide inflammation triggered by infection and is a major public health problem. Diet plays a vital role in immune health but its association with sepsis in humans is unclear.

**Methods:**

We examined 21,404 participants with available dietary data from the Reasons for Geographic and Racial Differences in Stroke (REGARDS) study, a national cohort of 30,239 black and white adults ≥45 years of age living in the US. The primary exposures of interest were five empirically derived diet patterns identified via factor analysis within REGARDS participants: “Convenience” (Chinese and Mexican foods, pasta, pizza, other mixed dishes), “Plant-based” (fruits, vegetables), “Southern” (added fats, fried foods, organ meats, sugar-sweetened beverages), “Sweets/Fats” (sugary foods) and “Alcohol/Salads” (alcohol, green-leafy vegetables, salad dressing). The main outcome of interest was investigator-adjudicated first hospitalized sepsis events.

**Results:**

A total of 970 first sepsis events were observed over ~6 years of follow-up. In unadjusted analyses, greater adherence to Sweets/Fats and Southern patterns was associated with higher cumulative incidence of sepsis, whereas greater adherence to the Plant-based pattern was associated with lower incidence. After adjustment for sociodemographic, lifestyle and clinical factors, greater adherence to the Southern pattern remained associated with higher risk of sepsis (hazard ratio [HR] comparing the fourth to first quartile, HR 1.39, 95 % CI 1.11,1.73). Race modified the association of the Southern diet pattern with sepsis (*P*_interaction_ = 0.01), with the Southern pattern being associated with modestly higher adjusted risk of sepsis in black as compared to white participants (HR comparing fourth vs. first quartile HR 1.42, 95 % CI 0.75,2.67 vs. 1.21, 95 % CI 0.93,1.57, respectively).

**Conclusion:**

A Southern pattern of eating was associated with higher risk of sepsis, particularly among black participants. Determining reasons for these findings may help to devise strategies to reduce sepsis risk.

**Electronic supplementary material:**

The online version of this article (doi:10.1186/s12879-015-0981-1) contains supplementary material, which is available to authorized users.

## Background

Sepsis is the syndrome of body-wide systemic inflammation triggered by severe microbial infection and is responsible for over 750,000 hospitalizations and 215,000 deaths in the US annually [1]. While considerable attention has focused on the factors that accelerate the progression and outcomes of acute sepsis in hospitalized patients, only limited data describe an individual’s propensity for developing sepsis [1–8].

Nutrition may play an important role in influencing sepsis risk in the community. Studies have shown that animals fed high-fat diets meant to mimic Western patterns of eating developed impaired immune function, inflammation, accelerated organ damage and death after induction of sepsis [9–11]. Population-based studies have shown that greater adherence to a Westernized diet was associated with increased biomarkers of inflammation and endothelial dysfunction [12–14], both of which have been implicated in the pathogenesis of sepsis [15–17]. Despite these plausible connections, few studies have examined the associations between diet and sepsis risk in a large, well-characterized cohort of community-dwelling adults.

Foods consist of a variety of nutrients that interact biologically and have important synergistic effects [18]. To better capture these relationships, diet patterns have been widely utilized to assess the impact of general patterns of food consumption (instead of or in addition to individual macro- or micronutrient intakes) on health outcomes [18, 19]. Dietary patterns can be examined using *a priori* dietary scores derived from pre-defined patterns of eating behavior (e.g., Healthy Eating Index, Mediterranean Diet Score, etc.) or by using *a posteriori*, data-driven methods [18]. This latter methodology has the advantage of not making any assumptions about diet quality based upon contemporary notions of diet-disease relationships but instead describes patterns of food consumption based on actual foods consumed within a particular population. We have previously identified five empirically derived diet patterns within the Reason for Geographic and Racial Differences in Stroke (REGARDS) study, a large national cohort of black and white adults in the United States [20]. Further, we reported independent associations of several of these patterns with stroke risk and mortality [20, 21]. Since stroke and sepsis share similar risk factors [22], the focus of this study was to examine the relationships between diet patterns and sepsis risk in REGARDS participants.

## Methods

### Study population and participants

The REGARDS study is a population-based investigation of stroke incidence in black and white US adults ≥45 years of age. Details of the study design have been reviewed elsewhere [23]. Briefly, the study was designed to provide approximately equal representation of men and women and oversampled individuals who were black as well as individuals living in eight Southeastern US states that have disproportionately high stroke mortality, termed the US stroke belt/buckle (North Carolina, South Carolina, Georgia, Tennessee, Mississippi, Alabama, Louisiana, and Arkansas). Trained personnel conducted computer-assisted telephone interviews to obtain information including participants’ sociodemographics, cardiovascular risk factors, cigarette smoking, physical activity, and use of medications. Following this call, trained health professionals conducted an in-home study visit that included an electrocardiograph (ECG) recording, blood pressure, height and weight measurements, inventory of medications and collection of blood and urine samples. Several questionnaires, including the 1998 Block food frequency questionnaire (Block 98 FFQ, NutritionQuest, Berkeley, CA), were left with participants to be completed after the in-home visit and mailed back to the study center. Overall, 30,239 black and white adults were enrolled between January 2003 and October 2007. The REGARDS study protocol was approved by the Institutional Review Boards (IRBs) governing research in human subjects at participating centers, and all participants provided informed consent. The University of Alabama at Birmingham served as the primary IRB of record for the study. The study was also approved by the University of Vermont, Wake Forest University, and University of Cincinnati.

### Diet assessment

Diet data were collected using the Block 98 FFQ, a semi-quantitative, 110-item FFQ that assessed a person’s usual diet over the past year, including frequency of consumption (average times per day, week, or month) and the portion size of specific foods or beverages (e.g., ½ cup of carrots, 2 slices of bacon) [24]. FFQs received by the study center were checked for completeness and scanned. Scanned files were then sent to NutritionQuest (http://www.nutritionquest.com) for analysis of nutrient contents using proprietary algorithms.

### Primary exposures

The exposures of interest were empirically derived diet pattern scores. Food and beverage questions from the FFQ were collapsed into 56 investigator-defined individual food groups. A principal components analysis (PCA) was used to derive diet patterns and factor loadings for each of the 56 individual food groups, as described in detail elsewhere [20]. The retained patterns (Convenience, Plant-based, Sweets/Fats, Southern, Alcohol/Salads) were named according to the highest food group loadings within each factor. In general, the Convenience pattern was characterized by high factor loadings for Chinese and Mexican food, pasta dishes, pizza, soup and other mixed dishes including frozen or take-out meals; the Plant-based pattern by fruits, vegetables, and fish; the Sweets/Fats pattern by desserts and other sugary foods; the Southern pattern by added fats, organ meats, fried foods, processed meats, sugar-sweetened beverages and greens typical of southern cuisines; and the Alcohol/Salads pattern by alcohol, green leafy vegetables, tomatoes, and salad dressing. A factor score for each of the patterns was calculated for each study participant by summing observed intakes of component food groups weighted by their respective factor loadings. Factor analysis differs from cluster analysis in that individuals may adhere to more than one dietary pattern identified in this analysis [18].

### Ascertainment of outcome

The outcome of interest was sepsis, ascertained via medical record review as described previously [22]. Briefly, trained research staff retrieved and reviewed medical records for all hospital admissions and emergency department visits attributed to a serious infection. Two abstractors independently reviewed all relevant clinical and laboratory data to confirm the presence of a serious infection on initial hospital presentation and the fulfillment of sepsis criteria. The abstractors adjudicated discordances, with additional physician-level review as needed.

Consistent with international consensus definitions [25], sepsis was defined as a presentation to the hospital with a serious infection plus two or more systemic inflammatory response criteria, including (1) heart rate > 90 beats/minute, (2) fever or hypothermia (temperature >38.3 °C or <36 °C), (3) tachypnea (>20 breaths/minute) or pCO_2_ < 32 mmHg, and (4) leukocytosis or leukopenia (white blood cells >12,000 or <4,000 cells/mm^3^ or >10 % band forms). A serious infection was defined according to a previously published taxonomy [1]. Vital status was determined based upon medical chart review. Because of our focus on community-acquired (vs. hospital acquired) sepsis, we utilized the worst diagnostic and laboratory values appearing during the first 28 h of hospitalization, allowing for 4 h of emergency department care plus 24 h of hospitalization. If a participant had more than one sepsis event during follow-up, then we selected the first event for analysis. Classification of sepsis was *not* based upon ICD-9 discharge diagnoses.

### Covariates of interest

Age, race, sex, smoking history, education and annual family income were determined by self-report. Physical activity was assessed through a single question: “How many times per week do you engage in intense physical activity, enough to work up a sweat?” Participants reported weekly television or video watching frequency on a written survey administered during the initial in-person examination with the following possible answers: none, 1–6 h/week, 1 h/day, 2 h/day, 3 h/day, and 4+ hours/day. Abdominal obesity was defined as waist circumference >88 cm for women and >102 cm for men. Hypertension was defined as a systolic blood pressure ≥140 mmHg and/or a diastolic blood pressure ≥90 mmHg, or a self-report of a prior diagnosis of hypertension or current use of anti-hypertensive medications. History of coronary heart disease (CHD) was defined as having any of the following: ECG evidence of myocardial infarction, prior history of a cardiac procedure (coronary artery bypass surgery or percutaneous coronary intervention), or self-reported history of myocardial infarction. Diabetes was as a fasting blood glucose concentration of ≥126 mg/dL, or a non-fasting blood glucose concentration of ≥200 mg/dL, or self-reported use of insulin or oral hypoglycemic agents. Chronic kidney disease (CKD) was defined as an estimated glomerular filtration rate <60 ml/min/1.73 m^2^ or a spot urine albumin to creatinine ratio ≥30 mg/g. REGARDS did not collect information on pulmonary conditions, and therefore we defined chronic lung disease as participant use of pulmonary medications including beta agonists, leukotriene inhibitors, inhaled corticosteroids, combination inhalers, and other pulmonary medications such as ipratropium, cromolyn, aminophylline and theophylline.

### Statistical analyses

Follow-up time for each participant was calculated from the date of the in-home visit to the date of death, sepsis or last telephone follow-up, updated through December 31^st^, 2012. Descriptive statistics were used to compare baseline characteristics of participants across quartiles of each diet pattern. The Kaplan-Meier method was used to calculate cumulative incidence of sepsis by quartile of each diet pattern, separately. Next, after confirming the proportionality of hazards, Cox regression models were used to estimate the hazard ratio of sepsis as a function of each diet pattern, separately, in sequential models. Model 1 was adjusted for age (continuous), race (black vs. white), sex (male vs. female), geographic region of residence (within vs. outside the Southeastern US defined as residence in stroke belt/buckle states), and total energy intake (continuous). Model 2 was adjusted for variables in Model 1 plus waist circumference, lifestyle factors (self-reported frequency of exercise per week [none, 1 to 3 times per week, or 4 or more times per week], television viewing [none, <4 h per day, 4 or more hours per day], smoking [current, past, or never]), comorbidities (history of heart disease, hypertension, diabetes, chronic pulmonary disease, CKD [all yes vs. no]), annual family income (<$20,000, $20,000–$50,000 or > $50,000/year) and educational achievement (< high school diploma, high school education, >high school education). Cut-points for categorized variables were based upon thresholds used in prior studies [21, 26]. Covariates were selected based on whether they are plausibly related biologically to the diet patterns and with the outcomes of interest based on existing literature. In all Cox models, diet patterns were analyzed in quartiles (with the lowest quartile serving as the referent group) and on a continuous scale. In pre-specified analyses, we examined for effect modification by age, race, sex and diabetes by examining the statistical significance of multiplicative interaction terms in multivariable models. Due to the time lag in observations and medical record retrieval, we could not review medical records for a portion of participants with reported hospitalizations for serious infection. In a sensitivity analysis, we repeated the analyses excluding these individuals. A two-tailed *P* value <0.05 was considered statistically significant for all analyses, except for tests of statistical interaction (*P* value <0.10). All analyses were conducted using SAS software version 9.3 (SAS Institute, Cary, NC).

## Results

### Study population

Of the 30,329 REGARDS participants, we excluded 288 participants missing follow-up data, 1,037 with implausible energy intake (defined as <500 or >4500 kcal/day for women and <800 or >5000 kcal/day for men), 4,919 who did not return an FFQ, and 2,591 who completed less than 85 % of the FFQ, leaving a total of 21,404 participants in the analyzed study sample.

Greater adherence to the Convenience and Alcohol/Salads patterns (defined by a greater proportion of participants in the fourth quartile as compared to the first) was associated with younger age, white race, male sex, residence outside the Southeast US, higher income and education, and lower prevalence of diabetes (Table 1). Higher adherence to the Convenience pattern was also associated with lower prevalence of CHD, hypertension and CKD, whereas higher adherence to the Alcohol/Salads was associated with higher prevalence of these conditions. Greater adherence to the Plant-based pattern was associated with older age, black race, female sex, non-smoking, greater physical activity, less sedentary behavior, and higher prevalence of diabetes and hypertension. Adherence to the Sweets/Fats pattern was greater among individuals who were white, men, residents of the Southeast US, current smokers, had lower abdominal adiposity, were less physically active and had greater sedentary behavior. Greater adherence to the Southern pattern was associated with younger age, black race, male sex, lower income and education, residence in the Southeast US, current smoking, higher abdominal adiposity, lower physical activity, greater sedentary behavior and higher prevalence of diabetes, CHD, hypertension and CKD.

**Table 1:** Participant characteristics by quartiles (Q) of diet pattern scores. Results are depicted as mean (standard error) or frequencies

		Q1	Q2	Q3	Q4
Convenience	Age, mean (SE)	67.3 (0.1)	65.4 (0.1)	64.4 (0.1)	62.3 (0.1)*
	Black (%)	46	37	28	23*
	Male (%)	35	39	48	55*
	Residence in the US Southeast (%)^a^	62	57	55	51*
	Annual family income < $20,000 (%)	24	18	15	14*
	Less than high school education (%)	14	10	7	7*
	Current Smoker (%)	13	14	13	15
	Abdominal adiposity (%)	48	47	46	48
	Physical activity (none) (%)	34	33	31	33
	Sedentary behavior (%)^b^	32	30	29	31*
	Diabetes (%)	22	20	17	18*
	Coronary heart disease (%)	19	17	16	15*
	Hypertension (%)	62	59	55	52**
	Chronic pulmonary disease (%)	9	9	9	10
	Chronic kidney disease (%)	23	21	19	17*
Plant Based	Age, mean (SE)	62.7 (0.1)	65.2 (0.1)	65.8 (0.1)	65.8 (0.1)*
	Black (%)	27	34	35	38*
	Male (%)	53	45	41	37*
	Residence in the US Southeast (%)^a^	56	58	56	55
	Annual family income < $20,000 (%)	18	18	18	18
	Less than high school education (%)	10	11	9	8*
	Current Smoker (%)	23	14	10	8*
	Abdominal adiposity (%)	47	48	47	47
	Physical activity (none) (%)	40	35	30	26*
	Sedentary behavior (%)^b^	36	32	29	26*
	Diabetes (%)	17	20	20	20*
	Coronary heart disease (%)	17	18	17	17
	Hypertension (%)	54	58	58	57**
	Chronic pulmonary disease (%)	9	9	10	10
	Chronic kidney disease (%)	19	22	20	19
Sweets/Fats	Age, mean (SE)	64.3 (0.1)	65.5 (0.1)	65.3 (0.1)	64.5 (0.1)
	Black (%)	45	33	28	28*
	Male (%)	38	41	47	49*
	Residence in the US Southeast (%)^a^	53	55	56	61*
	Annual family income < $20,000 (%)	18	17	16	20
	Less than high school education (%)	9	9	9	11**
	Current Smoker (%)	12	12	13	17*
	Abdominal adiposity (%)	50	47	46	46*
	Physical activity (none) (%)	31	32	33	36*
	Sedentary behavior (%)^b^	28	28	29	37*
	Diabetes (%)	22	21	19	16*
	Coronary heart disease (%)	16	18	17	18
	Hypertension (%)	59	57	56	55*
	Chronic pulmonary disease (%)	9	9	10	10**
	Chronic kidney disease (%)	21	20	20	19
Southern	Age, mean (SE)	64.9 (0.1)	65.2 (0.1)	65.5 (0.1)	63.9 (0.1)*
	Black (%)	9	24	41	60*
	Male (%)	37	39	46	54*
	Residence in the US Southeast (%)^a^	49	54	59	64*
	Annual family income < $20,000 (%)	10	14	20	27*
	Less than high school education (%)	4	7	11	17*
	Current Smoker (%)	8	11	15	21*
	Abdominal adiposity (%)	37	46	50	56*
	Physical activity (none) (%)	28	33	34	36*
	Sedentary behavior (%)^b^	22	26	33	42*
	Diabetes (%)	11	17	21	28*
	Coronary heart disease (%)	15	18	18	18*
	Hypertension (%)	46	54	61	67*
	Chronic pulmonary disease (%)	10	9	10	9
	Chronic kidney disease (%)	15	19	22	24*
Alcohol/Salads	Age, mean (SE)	66.3 (0.1)	65.2 (0.1)	64.5 (0.1)	63.6 (0.1)*
	Black (%)	50	38	26	19*
	Male (%)	36	40	47	53*
	Residence in the US Southeast (%)^a^	59	59	57	51*
	Annual family income < $20,000 (%)	30	19	14	9*
	Less than high school education (%)	16	11	7	5*
	Current Smoker (%)	11	13	15	15*
	Abdominal adiposity (%)	49	49	45	46**
	Physical activity (none) (%)	36	34	31	29*
	Sedentary behavior (%)^b^	35	32	28	27*
	Diabetes (%)	21	21	18	16*
	Coronary heart disease (%)	15	18	18	18*
	Hypertension (%)	46	54	61	67*
	Chronic pulmonary disease (%)	10	9	10	9
	Chronic kidney disease (%)	16	19	22	24*

*P test for trend < 0.001; **P test for trend < 0.05; linear test for trend was based on general linear regression with diet pattern score (continuous) as the independent variable. The Kruskal-Wallis test was used to generate *p*-values for categorical test for trend

^a^Defined as living in US stroke belt/buckle states (North Carolina, South Carolina, Georgia, Tennessee, Mississippi, Alabama, Louisiana, or Arkansas) at the time of the baseline visit; ^b^Defined as ≥ 4 h of television viewing/day

### Diet patterns and nutrient characteristics

The nutrient composition of each dietary pattern is depicted in Additional file 1: Table S1. Participants with greater adherence to the Convenience, Sweets/Fats, and Alcohol/Salads patterns consumed higher amounts of total fat and saturated fat as a percentage of total energy intake (Additional File 1: Table S1). Higher consumption of Convenience and Alcohol/Salads patterns was also associated with higher intake of protein as a percentage of total energy intake and higher intake of energy-adjusted mean sodium and potassium per day, whereas higher consumption of the Sweets/Fats pattern was associated with the opposite pattern. Higher adherence to the Southern pattern was characterized by greater energy intake from fats at the expense of protein and carbohydrates, and lower energy-adjusted mean fiber intake. Higher adherence to the Plant-based pattern was associated with lower percentage of energy intake from total fat and saturated fat, lower intake of *trans* fats, and higher intake of fiber.

### Diet patterns and risk of sepsis

**Fig. 1:**
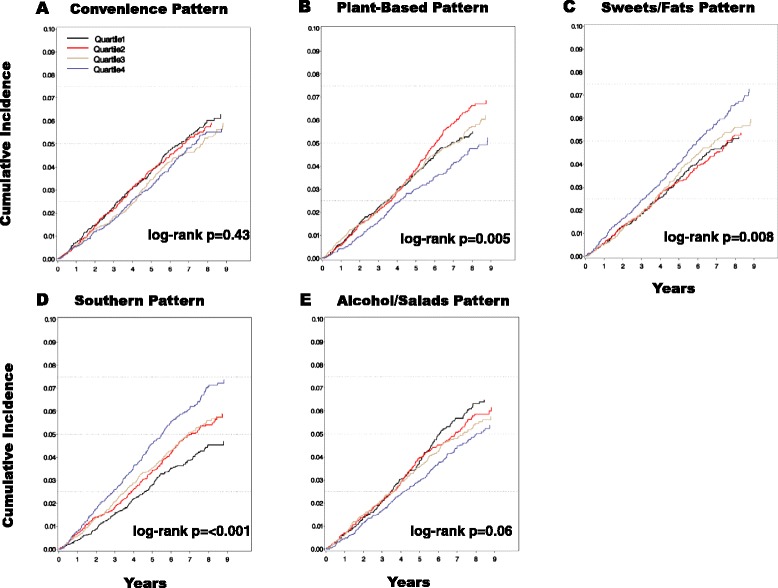
Kaplan-Meier curves of cumulative incidence of sepsis according to quartiles of **a** Convenience, **b** Plant-based, **c** Sweets/Fats, **d** Southern, and **e** Alcohol/Salads diet pattern scores

A total of 970 sepsis events were observed over a median 6.7 (interquartile range 5.3–8.1) years of follow-up. Infection types associated with hospitalizations for sepsis are tabulated in Table 2. Higher quartiles of the Convenience and Alcohol/Salads patterns were associated with lower cumulative incidence of sepsis, but these associations were not statistically significant (Fig. 1a and e). Higher quartiles of the Sweets/Fats and Southern patterns were associated with higher cumulative incidence of sepsis (*P*_log-rank_ = 0.008 and <0.001, respectively, Fig. 1c and d), whereas the highest Plant-based pattern quartile was associated with lower cumulative incidence of sepsis than the three lowest (*P*_log-rank_ = 0.005, Fig. 1b).

**Table 2:** Infection types associated with hospitalizations for sepsis

	Serious Infection type (%)
Pneumonia	42
Kidney/Genitourinary tract	15
Meningitis	0.4
Skin/soft tissue	7
Catheter related (IV/Central/Dialysis)	0.4
Intra-abdominal infection	16
Surgical Wound	0.8
Bronchitis, influenza and other lung infections	9
Unknown	9

There were no statistically significant associations of Convenience or Sweets/Fats patterns with adjusted hazard ratios (HR) of sepsis in any models (Table 3). Greater adherence to the Plant-based and Alcohol/Salads patterns were associated with lower risk of sepsis in models adjusted for age, race, sex, geographic region of residence and total energy intake (HRs comparing fourth to first quartile 0.68, 95 % confidence interval [CI] 0.56,0.83; and 0.78, 95 % CI 0.65,0.94, respectively). These associations were no longer statistically significant after further adjustment for sociodemographic characteristics, lifestyle factors, waist circumference and co-morbidities. There was a graded association of higher quartiles of the Southern pattern with higher risk of sepsis in models adjusted for age, race, sex, geographic region of residence and total energy intake (quartile 1 ref; quartile 2, HR 1.29, 95 % CI 1.07,1.56; quartile 3 HR 1.37, 95 % CI 1.13,1.66; quartile 4 HR 1.93, 95 % CI 1.57,2.36). After further adjustment for sociodemographic characteristics, lifestyle factors, waist circumference and co-morbidities, the highest quartile of the Southern pattern remained significantly associated with higher risk of sepsis as compared with the lowest (HR 1.39, 95 % CI 1.11,1.73). The results were qualitatively the same when the analysis was restricted to individuals with an annual income of > $20,000/year—as compared to individuals in the lowest quartile of the Southern diet pattern, individuals in higher quartiles of the Southern pattern had higher risk of sepsis in fully adjusted models (quartile 1 ref; quartile 2, HR 1.22, 95 % CI 0.97,1.55; quartile 3 HR 1.23, 95 % CI 0.97,1.57; quartile 4 HR 1.40, 95 % CI 1.08,1.83; *P*_trend_ = 0.02). The results did not change after excluding 428 participants who reported hospitalization for infection but whose medical records had not yet been retrieved for adjudication of possible sepsis events (data not shown).

**Table 3:** Hazard ratios of sepsis (95 % confidence intervals) as a function of diet pattern quartiles (Q)

		Q1	Q2	Q3	Q4	*P*_trend_
Convenience	Events	257	248	236	235	
	Model 1	ref	1.02 (0.86, 1.22)	0.93 (0.77, 1.11)	0.93 (0.75, 1.14)	0.33
	Model 2	ref	1.04 (0.86, 1.25)	0.97 (0.79, 1.17)	0.93 (0.74, 1.15)	0.42
Plant Based	Events	236	281	254	205	
	Model 1	ref	1.05 (0.88, 1.25)	0.90 (0.75, 1.08)	0.68 (0.56, 0.83)	<0.001
	Model 2	ref	1.22 (1.01, 1.47)	1.09 (0.89, 1.33)	0.89 (0.72, 1.12)	0.23
Sweets/Fats	Events	221	225	244	286	
	Model 1	ref	0.90 (0.75, 1.09)	0.97 (0.80, 1.17)	1.13 (0.91, 1.39)	0.24
	Model 2	ref	0.79 (0.65, 0.98)	0.91 (0.74, 1.11)	1.01 (0.81, 1.27)	0.69
Southern	Events	200	247	233	296	
	Model 1	ref	1.29 (1.07, 1.56)	1.37 (1.13, 1.66)	1.93 (1.57, 2.36)	<0.001
	Model 2	ref	1.20 (0.99, 1.47)	1.11 (0.89, 1.37)	1.39 (1.11, 1.73)	0.009
Alcohol/Salads	Events	265	250	243	218	
	Model 1	ref	0.96 (0.80, 1.14)	0.89 (0.75, 1.07)	0.78 (0.65, 0.94)	0.009
	Model 2	ref	0.93 (0.77, 1.12)	0.91 (0.75, 1.10)	0.84 (0.69, 1.03)	0.11

Model 1 adjusted for age, race, sex, geographic region of residence, and energy intake

Model 2 adjusted for variables in Model 1 plus waist circumference, annual income, educational achievement, physical activity, sedentary behavior, current smoking, hypertension, diabetes, coronary artery disease, chronic pulmonary disease and chronic kidney disease

The magnitude and strength of these associations did not differ by sex, age (< vs. ≥64 years of age), or diabetes (*P*_interaction_ >0.10 for all). However, the association of Southern pattern scores with risk of sepsis differed by race (*P*_interaction_ = 0.01). In fully adjusted models, the risk of sepsis comparing the highest to lowest quartile of Southern pattern was numerically greater in black than in white participants (HR 1.42, 95 % CI 0.75,2.67 among black participants vs.HR 1.21, 95 % CI 0.93,1.57 among white participants).

## Discussion

In this large national cohort, higher scores for a diet pattern characterized by fried foods, processed meats, and sugar-sweetened beverages—foods commonly found in Southern cuisines—were independently associated with higher long-term risk of sepsis, particularly among individuals of black race. To our knowledge, this is the first study to link diet patterns with sepsis risk in a large US cohort, underscoring the important influence of diet on sepsis.

There are plausible connections between diet patterns and long-term sepsis risk. While malnutrition is the most important cause of nutrition-related immune deficiency worldwide [27], excess energy intake and obesity are increasingly recognized mediators of immune dysfunction [28]. Experimental studies have shown that animals fed high-energy, high-fat diets develop immune deficiencies and increased susceptibility to infectious disease and sepsis [9–11]. The mechanisms for these findings appear to be related to impairment of innate immune systems key to combating microbial infection [29]. For example, animals fed high fat diets had reduced numbers of phagocytic granulocytes, leading to an impaired ability to clear microbial infection and survive sepsis. In addition, animals fed high fat diets develop excess inflammatory cytokine production in response to infectious stimuli. Importantly, however, these studies were conducted in mice and may not be directly applicable to human disease. The few human data that are available come from population-based studies which have consistently shown an association of Westernized patterns of eating with key mediators of sepsis such as inflammation and endothelial cell activation [12–14], whereas healthy diets replete with fruits and vegetables have been associated with the opposite [30, 31]. The results of the current study add to these data by showing that a diet pattern characterized by high intake of fried, fatty foods, processed meats and sugar-sweetened beverages is associated with increased risk of sepsis in community-dwelling adults independently of key confounders such as diabetes, abdominal obesity, and sedentary behavior.

Most clinical and scientific attention have focused on the factors that accelerate the progression of acute sepsis as well as methods for its acute resuscitation [1–8]. However, only limited data describe the baseline characteristics that heighten an individual’s risk of developing a future sepsis event. Thus, the finding of an association between the Southern diet pattern and sepsis risk in the current study is important because it suggests that targeted efforts to reduce the intake of processed and fried food and sugar-sweetened beverages may represent a novel intervention for reducing sepsis risk in high-risk populations.

Black participants exhibited much higher adherence to the Southern diet pattern than white participants. This is important in that the magnitude of the association between the Southern pattern and sepsis appeared to be greater in black than in white participants. When coupled with recent data from our group showing that the Southern diet pattern explained a substantial portion of the excess risk of stroke among black participants of REGARDS [20], these findings collectively support the notion that regional and/or cultural patterns of eating deserve closer scrutiny in understanding racial disparities in health outcomes.

Our study also had limitations. As is the case for any study relying on FFQ data, dietary reporting errors may have reduced the accuracy of individual dietary intake measurements and resulted in misclassification [32]. However, this would generally bias results towards the null, potentially reducing the magnitude of the observed associations between diet patterns and sepsis. Next, roughly one third of the cohort did not return the FFQ. Those who did not return the FFQ were more likely to have annual incomes less than $20,000 and less than a high school education [24]; however, the ~900 people of lower socioeconomic status who did return the FFQ were similar in terms of race, age, sex or history of cardiovascular disease from those who did not return the FFQ, minimizing the likelihood of substantial bias. Although we adjusted for available lifestyle factors such as tobacco use and sedentary behavior, we cannot exclude residual confounding from other unmeasured lifestyle factors strongly linked with diet. In addition, the REGARDS Study was not specifically designed to examine the association of diet and sepsis risk.

## Conclusions

In conclusion, greater adherence to a Southern diet pattern characterized by high intake of fried foods, processed meats, and sugar-sweetened beverages and by disproportionately high residence in the Southeastern US, was independently associated with increased sepsis risk. Studies elucidating whether these observed associations are causal may be warranted.

## Additional file

Additional file 1: Table S1. Nutrient intakes by quartile (Q) of diet pattern scores. Results are depicted as mean (standard error). Intakes of each nutrient are adjusted for total energy intake except for percent energy intake from fat, protein, carbohydrates, and saturated fat.

## Abbreviations

eGFR: Estimated glomerular filtration rate
ACR: Albumin to creatinine ratio
REGARDS: Reasons for Geographic and Racial Differences in Stroke
FFQ: Food frequency questionnaire
