# Supplementary material for: Diet patterns and risk of sepsis in community-dwelling adults: a cohort study
Source: BMC Infect Dis. 2015 Jun 14;15:231. doi: 10.1186/s12879-015-0981-1 (PMC4465736; doi:10.1186/s12879-015-0981-1)
Supplement: Additional file 1: Table S1. — Nutrient intakes by quartile (Q) of diet pattern scores. Results are depicted as mean (standard error). Intakes of each nutrient are adjusted for total energy intake except for percent energy intake from fat, protein, carbohydrates, and saturated fat. [file 12879_2015_981_MOESM1_ESM.docx]

**Additional File**

**Additional Table 1**. Nutrient intakes by quartile (Q) of diet pattern scores. Results are depicted as mean (standard error). Intakes of each nutrient are adjusted for total energy intake except for percent energy intake from fat, protein, carbohydrates, and saturated fat.

|  |  | Q1 | Q2 | Q3 | Q4 |
| --- | --- | --- | --- | --- | --- |
| **Convenience** | Energy, kJ | 1428 (18) | 1405 (18) | 1630 (19) | 2200 (25) ^*^ |
|  | % Total fat | 36.2 (0.3) | 36.5 (0.2) | 37.4 (0.2) | 38.2 (0.2) ^*^ |
|  | % Saturated Fat | 10.5 (0.1) | 10.6 (0.1) | 10.7 (0.1) | 11.1 (0.1) ^*^ |
|  | % Protein | 13.0 (0.1) | 14.2 (0.1) | 14.9 (0.1) | 15.9 (0.1) ^*^ |
|  | % Carbohydrates | 52.4 (0.3) | 49.1 (0.3) | 47.9 (0.3) | 46.1 (0.2) ^*^ |
|  | % Alcohol | 1.6 (0.1) | 2.8 (0.2) | 2.7 (0.2) | 2.5 (0.2)^*^ |
|  | Polyunsaturated fatty acid, g | 18.6 (0.2) | 18.1 (0.2) | 18.2 (0.2) | 17.3 (0.2) ^*^ |
|  | Monounsaturated fatty acid, g | 25.4 (0.2) | 26.1 (0.2) | 26.5 (0.2) | 26.9 (0.2) ^*^ |
|  | *Trans* Fat, g | 5.7 (0.1) | 5.9 (0.1) | 5.9 (0.1) | 5.7 (0.1) |
|  | Fiber, g | 15.1 (0.2) | 14.8 (0.2) | 15.4 (0.2) | 16.0 (0.2) ^*^ |
| **Plant Based** | Energy, kJ | 1490 (22) | 1484 (19) | 1635 (20) | 2049 (25) ^*^ |
|  | % Total fat | 38.5 (0.2) | 37.5 (0.2) | 37.1 (0.2) | 35.4 (0.2) ^*^ |
|  | % Saturated Fat | 11.7 (0.1) | 11.0 (0.1) | 10.5 (0.1) | 9.7 (0.1) ^*^ |
|  | % Protein | 13.5 (0.1) | 14.3 (0.1) | 14.7 (0.1) | 15.4 (0.1) ^*^ |
|  | % Carbohydrates | 46.8 (0.1) | 48.6 (0.2) | 49.5 (0.3) | 51.2 (0.3) ^*^ |
|  | % Alcohol | 4.0 (0.2) | 2.4 (0.2) | 1.8 (0.1) | 1.3 (0.1)^*^ |
|  | Polyunsaturated fatty acid, g | 17.6 (0.2) | 18.1 (0.2) | 18.7 (0.2) | 17.8 (0.2) |
|  | Monounsaturated fatty acid, g | 27.8 (0.2) | 26.9 (0.2) | 26.2 (0.2) | 23.9 (0.2) ^*^ |
|  | *Trans* Fat, g | 6.6 (0.1) | 6.3 (0.1) | 5.8 (0.1) | 4.6 (0.1) ^*^ |
|  | Fiber, g | 10.8 (0.2) | 13.3 (0.2) | 15.9 (0.2) | 21.3 (0.2) ^*^ |
| **Sweets/Fats** | Energy, kJ | 1250 (18) | 1395 (15) | 1679 (16) | 2337 (23) ^*^ |
|  | % Total fat | 34.7 (0.3) | 36.7 (0.2) | 37.9 (0.2) | 39.2 (0.2) ^*^ |
|  | % Saturated Fat | 9.9 (0.1) | 10.6 (0.1) | 10.9 (0.1) | 11.4 (0.1) ^*^ |
|  | % Protein | 15.9 (0.1) | 14.6 (0.1) | 14.2 (0.1) | 13.2 (0.1) ^*^ |
|  | % Carbohydrates | 48.4 (0.3) | 49.5 (0.3) | 49.6 (0.3) | 48.9 (0.3) ^**^ |
|  | % Alcohol | 4.6 (0.3) | 2.3 (0.2) | 1.4 (0.1) | 1.2 (0.1) ^*^ |
|  | Polyunsaturated fatty acid, g | 17.0 (0.2) | 17.9 (0.2) | 18.2 (0.2) | 19.1 (0.2) ^*^ |
|  | Monounsaturated fatty acid, g | 25.4 (0.2) | 25.9 (0.2) | 26.6 (0.2) | 27.1 (0.2) ^*^ |
|  | *Trans* Fat, g | 4.5 (0.1) | 5.2 (0.1) | 5.9 (0.1) | 7.6 (0.1) ^*^ |
|  | Fiber, g | 16.4 (0.2) | 16.2 (0.2) | 15.6 (0.2) | 13.2 (0.2) ^*^ |
| **Southern** | Energy, kJ | 1567 (19) | 1377 (17) | 1584 (19) | 2144 (26) ^*^ |
|  | % Total fat | 35.3 (0.2) | 36.9 (0.2) | 37.5 (0.2) | 38.8 (0.2) ^*^ |
|  | % Saturated Fat | 10.0 (0.1) | 10.6 (0.1) | 10.9 (0.1) | 11.4 (0.1) ^*^ |
|  | % Protein | 15.0 (0.1) | 14.4 (0.1) | 14.1 (0.1) | 14.4 (0.1) ^**^ |
|  | % Carbohydrates | 50.7 (0.3) | 49.6 (0.3) | 48.9 (0.3) | 47.0 (0.3) ^*^ |
|  | % Alcohol | 2.7 (0.2) | 2.3 (0.2) | 2.4 (0.2) | 2.2 (0.2) ^**^ |
|  | Polyunsaturated fatty acid, g | 17.4 (0.2) | 18.4 (0.2) | 18.3 (0.2) | 18.1 (0.2) ^*^ |
|  | Monounsaturated fatty acid, g | 25.5 (0.2) | 26.6 (0.2) | 26.6 (0.2) | 26.3 (0.2) ^*^ |
|  | *Trans* Fat, g | 5.7 (0.1) | 5.9 (0.1) | 5.9 (0.1) | 5.6 (0.1) |
|  | Fiber, g | 18.3 (0.2) | 16.1 (0.2) | 14.7 (0.2) | 12.2 (0.2) ^*^ |
| **Alcohol/Salads** | Energy, kJ | 1595 (23) | 1483 (19) | 1610 (19) | 1969 (25) ^*^ |
|  | % Total fat | 33.2 (0.2) | 36.4 (0.2) | 38.1 (0.2) | 40.8 (0.3) ^*^ |
|  | % Saturated Fat | 9.5 (0.1) | 10.5 (0.1) | 10.9 (0.1) | 11.8 (0.1) ^*^ |
|  | % Protein | 14.0 (0.1) | 14.2 (0.1) | 14.7 (0.1) | 14.9 (0.1) ^*^ |
|  | % Carbohydrates | 54.9 (0.3) | 51.1 (0.3) | 47.9 (0.3) | 42.4 (0.3) ^*^ |
|  | % Alcohol | 0.5 (0.1) | 0.9 (0.1) | 2.5 (0.2) | 5.5 (0.3) ^*^ |
|  | Polyunsaturated fatty acid, g | 15.8 (0.2) | 17.7 (0.2) | 18.6 (0.2) | 20.1 (0.2) ^*^ |
|  | Monounsaturated fatty acid, g | 24.0 (0.2) | 25.9 (0.2) | 26.6 (0.2) | 28.4 (0.2) ^*^ |
|  | *Trans* Fat, g | 6.3 (0.1) | 6.1 (0.1) | 5.7 (0.1) | 4.9 (0.1) ^*^ |
|  | Fiber, g | 16.4 (0.2) | 15.2 (0.2) | 15.2 (0.2) | 14.6 (0.2) ^*^ |

^*^*P* for linear trend < 0.001, ^**^*P* for linear trend < 0.05; test for trend based upon linear regression with diet pattern score (continuous) as the independent variable.
